# Transgene‐independent heredity of RdDM‐mediated transcriptional gene silencing of endogenous genes in rice

**DOI:** 10.1111/pbi.12934

**Published:** 2018-05-30

**Authors:** Yuhya Wakasa, Taiji Kawakatsu, Takeo Harada, Fumio Takaiwa

**Affiliations:** ^1^ Plant Molecular Farming Unit Institute of Agrobiological Sciences National Agriculture and Food Research Organization Tsukuba Japan; ^2^ Faculty of Agriculture and Life Science Hirosaki University Hirosaki Japan

**Keywords:** GM crop, new plant breeding techniques, *Oryza sativa* L., RNA‐directed DNA methylation, transcriptional gene silencing

## Abstract

To induce transcriptional gene silencing (TGS) of endogenous genes of rice (*Oryza sativa* L.), we expressed double‐strand RNA of each promoter region and thus induced RNA‐directed DNA methylation (RdDM). We targeted constitutively expressed genes encoding calnexin (*CNX*), protein disulphide isomerase (*PDIL1‐1*) and luminal binding protein (*BiP1*); an endoplasmic reticulum stress‐inducible gene (*OsbZIP50*); and genes with seed‐specific expression encoding α‐globulin (*Glb*‐*1*) and glutelin‐B4 (*GluB4*). TGS of four genes was obtained with high efficiency (*CNX*, 66.7% of regenerated plants; *OsBiP1*, 67.4%; *OsbZIP50*, 63.4%; *GluB4*, 66.1%), whereas the efficiency was lower for *PDIL1‐1* (33.3%) and *Glb‐1* TGS lines (10.5%). The heredity of TGS, methylation levels of promoter regions and specificity of silencing of the target gene were investigated in some of the TGS lines. In progeny of *CNX* and *OsbZIP50* TGS lines, suppression of the target genes was preserved (except in the endosperm) even after the removal of trigger genes (T‐DNA) by segregation. TGS of *CNX* was reverted by demethylation treatment, and a significant difference in CG and CHG methylation levels in the −1 to −250 bp region of the *CNX* promoter was detected between the TGS and revertant lines, suggesting that TGS is closely related to the methylation levels of promoter. TGS exhibited specific suppression towards the target gene compared with post‐transcriptional gene silencing when *GluB4* gene from glutelin multigene family was targeted. Based on these results, future perspectives and problems to be solved in the application of RdDM to new plant breeding techniques in rice are discussed.

## Introduction

Genetically modified (GM) crops are commercially cultivated in 28 countries worldwide. The total global cultivated area of transgenic crops in 2015 was estimated at approximately 179.7 million hectares (James, 2016). However, the use of conventional techniques such as electroporation, particle bombardment and *Agrobacterium*‐mediated transformation are still viewed as a serious issue in many countries because of gene flow and its influence on the environment.

Recently, new plant breeding techniques (NPBTs) have been suggested as a novel strategy for plant trait improvement; NPBTs are quite different from conventional GM techniques. Approaches such as genome editing (ZFN, TALEN, CRISPR/Cas9), *Agrobacterium* infiltration to transiently express gene, grafting on GM rootstocks, reverse breeding and RNA‐directed DNA methylation (RdDM) are classified as NPBTs (Schaart *et al*., 2016). Plants modified by NPBTs are difficult to distinguish from natural mutants, and RdDM of genomic DNA cannot be detected by conventional techniques such as PCR and DNA sequencing (Schaart *et al*., 2016).

RdDM is induced through the generation of 21‐ to 24‐nucleotide (nt) short interfering RNAs (siRNAs), which is part of plant defence response against DNA viruses (Pelaez and Sanchez, 2013). RdDM also suppresses activation of transposable elements in plants (Lisch, 2009). siRNAs induce RdDM of specific cytosine residues (CG, CHG and CHH; where H is A, C or T) in target DNA regions, resulting in transcriptional gene silencing (TGS) (Lusser *et al*., 2012; Matzke *et al*., 2015). TGS of a target gene can be achieved by artificial introduction of double‐strand (ds)RNA corresponding to its promoter region, and these epigenetic changes are inherited. However, only a few reports describe transcriptional silencing of endogenous genes by RdDM and are inheritance (Kasai and Kanazawa, 2013). Most of the previous reports described TGS of transgenes such as green fluorescent protein (GFP) or β‐glucuronidase (GUS) reporter genes under the control of the CaMV 35S promoter (Kasai and Kanazawa, 2013). In rice (*Oryza sativa* L.), RdDM‐mediated TGS was induced to suppress the expression of the GFP gene under the control of the CaMV 35S promoter (35S::GFP), or seven endogenous genes (Okano *et al*., 2008). Expression of 35S::GFP was strongly suppressed by TGS, whereas only one endogenous gene was suppressed in spite of a high level of cytosine methylation in all target genes. Approximately 20% of the transcripts of the successfully silenced gene remained in comparison with its wild‐type level (Okano *et al*., 2008). TGS of endogenous genes induced by the expression of dsRNA corresponding to the promoter region using T‐DNA or viral vectors was reported in *Arabidopsis* (Bond and Baulcombe, 2015; Deng *et al*., 2014), petunia (Kanazawa *et al*., 2011), tomato (Kanazawa *et al*., 2011), potato (Heilersig *et al*., 2006; Kasai *et al*., 2016) and tobacco (Ju *et al*., 2016; Kon and Yoshikawa, 2014).

In this study, we aimed to induce RdDM‐mediated TGS of endogenous genes with different expression profiles (constitutive expression, endoplasmic reticulum [ER] stress‐inducible expression and seed‐specific expression) in rice. Several TGS rice lines were obtained and TGS was inherited until at least the third generation after the removal of the TGS trigger gene by segregation in some of these lines. These TGS lines lacking the trigger gene are epimutants, and our strategy to produce such a TGS rice line can be called epigenome editing. Our results show that RdDM‐mediated TGS may be useful as an NPBT in rice breeding, although some problems remain to be solved before its reliable application.

## Results

### Induction efficiency of TGS of endogenous genes in rice

**Figure 1:**
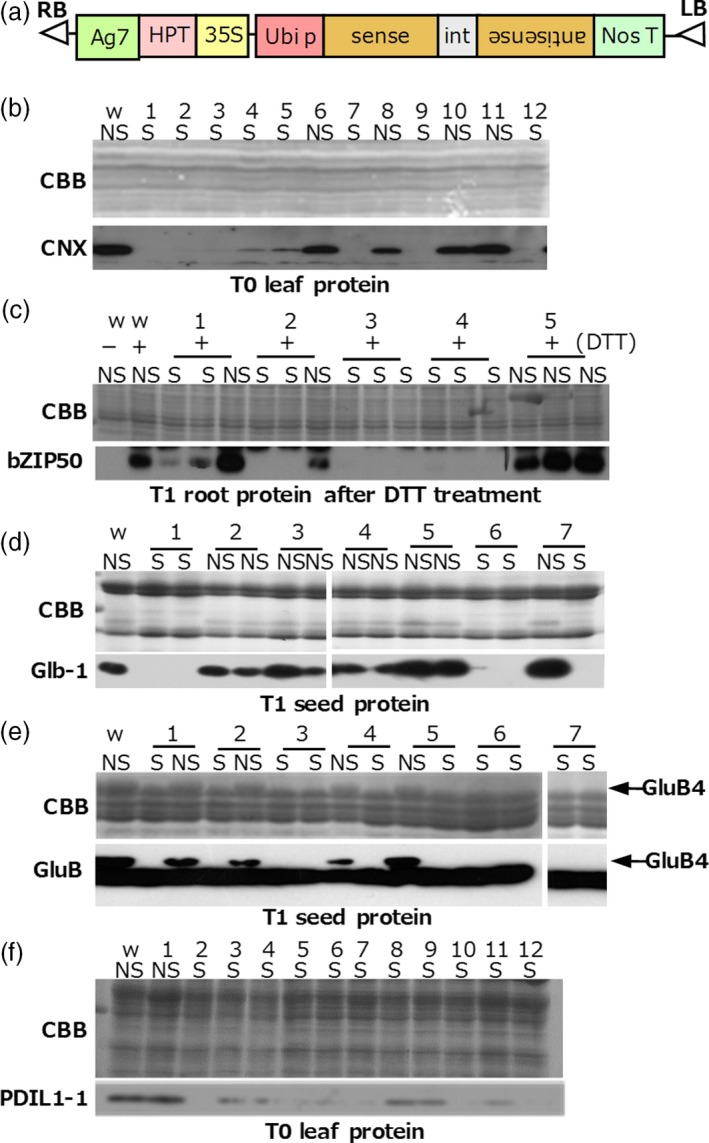
RdDM‐mediated TGS of endogenous genes in rice. (a) Binary vectors used to express the dsRNA to silence target genes. 35S, CaMV 35S promoter; HPT, hygromycin phosphotransferase; Ag7, Agrobacterium gene 7 terminator; Ubi p, rice ubiquitin promoter; int, 2nd intron of the rice aspartic protease (RAP) gene; NosT, nopaline synthase gene terminator; LB, left border; RB, right border. (b–f), Immunoblots of target proteins in total protein extracts from T0 leaves, T1 roots and T1 mature seed (as indicated) of TGS (S) and non‐TGS lines (NS). (b) CNX. (c) OsbZIP50; roots were either treated (+) or not (‐) with DTT to induce ER stress. (d) Glb‐1. (e), GluB4; GluB antibody reacts with GluB1, GluB2 and GluB4. Arrows show the position of GluB4 acidic subunit. (f) PDIL1‐1. CBB, Coomassie Brilliant Blue (loading control); w, wild type.

We constructed six binary vectors harbouring TGS trigger genes to induce TGS of calnexin (*CNX*), *OsbZIP50*, α‐globulin (*Glb‐1*), glutelin‐B4 (*GluB4*), protein disulphide isomerase (*PDIL1‐1*) and luminal binding protein (*OsBiP1*). Three of these genes (*CNX*,* PDIL1‐1* and *OsBiP1*) are constitutively expressed, the expression of *OsbZIP50* is induced by ER stress, and *GluB4* and *Glb‐1* are specifically expressed in the endosperm. To evaluate protein accumulation in TGS lines, we developed high‐quality antibodies against these proteins. To trigger TGS, we constitutively expressed dsRNA corresponding to an approximately 1‐kb promoter region of each gene under the control of the rice ubiquitin promoter (Figure 1a).

Because RdDM is a key factor for the artificial induction of TGS, methylation levels of the promoter regions of target genes were evaluated in transgenic rice plants harbouring the TGS trigger gene cassettes targeting *CNX*,* OsbZIP50* and *Glb‐1*. Genomic DNA digestion with the methylation‐sensitive restriction enzymes (*Hap*II, *Msp*I or *Hha*I) followed by PCR amplification of each restriction site showed clear signals in transgenic plants but not in the wild type, suggesting that the promoter regions of the target genes were methylated (Figure S1).

Immunoblotting analysis of protein samples prepared from young T0 leaves (for CNX and PDIL1‐1), mature T1 seed (Glb‐1 and GluB4) and young T1 roots subjected to ER stress (OsbZIP50) was performed to evaluate the efficiency of RdDM‐induced TGS. When the levels of target proteins were drastically decreased in comparison with those in the wild type, we defined these lines as TGS lines (Figure 1b–f). TGS lines were obtained at a high rate for *CNX* (66.7% of regenerated plants), *OsbZIP50* (63.4%) and *GluB4* (66.1%), and at a lower rate for *Glb‐1* (10.5%) and *PDIL1‐1* (33.3%). Many plants with the suppressed expression of *OsBiP1* withered during culture from regeneration medium to hormone‐free medium (Figure S2a), consistent with *OsBiP1* being an essential gene. We confirmed cytosine methylation of leaf DNA in very young regenerated plantlets before they withered (Figure S2b). A clear relationship was observed between poor growth and increased methylation levels (Figure S2b). Therefore, withered plantlets on hormone‐free medium were considered as TGS plants; 67.4% of regenerated plants were TGS plants. In *CNX* and *OsbZIP50* TGS lines, the results of analyses of methylation (Figure S1) and of TGS induction (Figure 1) were consistent with each other for each lane (compare lanes 1–9 in Figures S1b and Figure 1b, and lanes 1–5 in Figure S1c and Figure 1c). However, for *Glb‐1*, changes in the methylation level were detected in many transgenic lines, but only 6 of 57 regenerated plants were TGS lines (Figure S1d). Data on the six types of RdDM‐mediated TGS lines produced are summarized in Table 1.

**Table 1:** Induction efficiency of transcriptional gene silencing in six endogenous genes

Target gene	Expression profile	TGS plants/regenerated plants (%)
Calnexin (*CNX*)	Constitutive	44/66 (66.7%)
*OsbZIP50*	ER stress–inducible	26/41 (63.4%)
α‐Globulin (*Glb‐1*)	Endosperm‐specific	6/57 (10.5%)
Glutelin B4 (*GluB4*)	Endosperm‐specific	37/56 (66.1%)
Protein disulphide isomerase like 1‐1 (*PDIL1‐1*)	Constitutive	11/33 (33.3%)
Luminal binding protein (*OsBiP1*)	Constitutive + ER stress–inducible	a62/92 (67.4%)

a Plants with severely inhibited growth and withered plants were counted as TGS lines.

**Figure 2:**
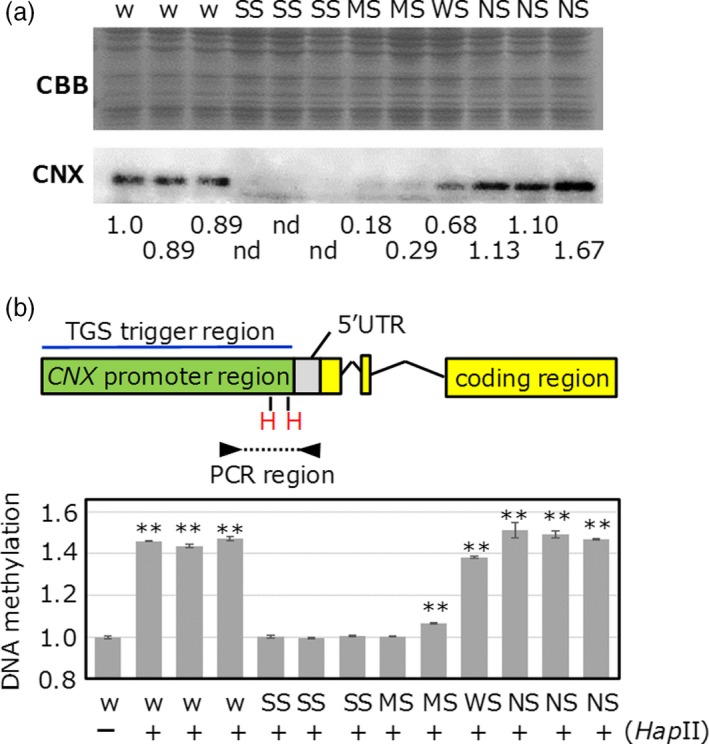
The relationship between the DNA cytosine methylation and TGS levels in *CNX* TGS lines. (a) Screening of various TGS lines. Wild type (w), strong TGS (SS), moderate TGS (MS), weak TGS (WS) and no TGS (NS) lines were screened. (b) Quantitative PCR after digestion of genomic DNA with methylation‐sensitive restriction enzyme *Hap* II (H) was shown as a bar graph. There are two *Hap* II sites between the primer sets. After overnight digestion with restriction enzymes, real‐time PCR amplification was performed. All PCR products were derived from endogenous genomic DNA but not from vector. (‐) shows undigested DNA sample; (+) shows digested DNA sample. Asterisks show statistically significant (*P < 0.05, **P < 0.01) differences (ΔCt values) relative to wt without *Hap* II digestion.

Because each transgenic line showed different levels of TGS (Figure 1), we quantitatively investigated the relationship between the TGS strength and methylation level within the *CNX* promoter in *CNX* TGS lines (Figure 2). TGS levels were divided into three categories, (i) strong (no signal was detected in immunoblot), (ii) moderate or weak (signal was detected but its signal was lower than that of nontransgenic) and (iii) no TGS (Figure 2a). To assess DNA methylation levels within the *CNX* promoter, we digested genomic DNA with methylation‐sensitive *Hap*II, which cannot digest methylated recognition sites, and amplified the promoter region (Figure 2b). Quantitative PCR revealed that relative DNA methylation levels of strong TGS lines and one moderate TGS line showed almost the same as genomic DNA without *Hap*II digestion, suggesting that almost two *Hap*II sites in PCR target region are highly methylated. DNA methylation levels of the other moderate TGS line were slightly higher than genomic DNA without *Hap*II digestion. On the other hand, DNA methylation levels in weak TGS line were slightly lower than wild type and no TGS lines, suggesting that *Hap*II sites in PCR target region were substantially unmethylated. No TGS lines showed very similar DNA methylation level to wild type. These results suggest that differences in methylation level in promoter region are associated with strength of TGS.

### Transgene‐independent heredity in some TGS lines

**Figure 3:**
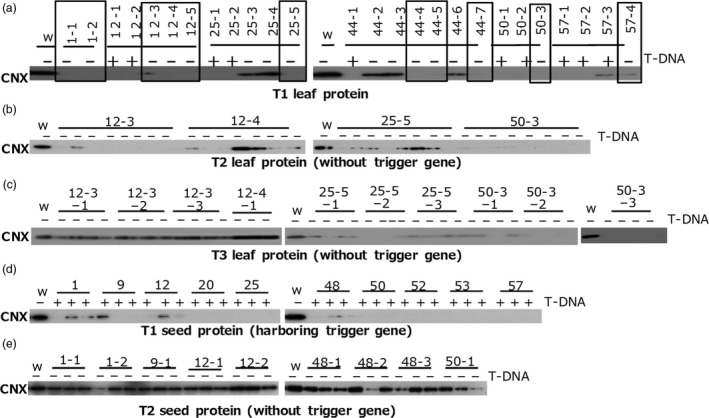
T‐DNA‐independent heredity of TGS in *CNX* TGS lines. CNX was detected by immunoblotting of total protein extracts from (a) T1 leaves of lines with (+) or without (‐) T‐DNA, (b) T2 leaves of lines without T‐DNA, (c) T3 leaves of lines without T‐DNA, (d) T1 seed of lines with T‐DNA and (e) T2 seed of lines without T‐DNA. Open squares in (a) indicate *CNX* TGS lines.

**Figure 4:**
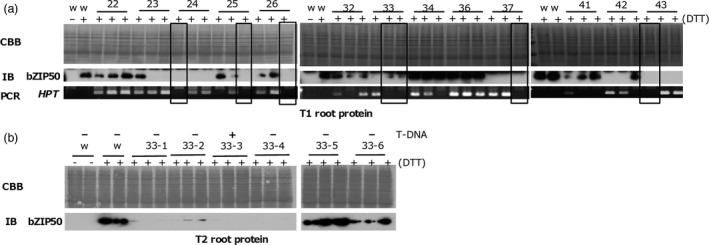
T‐DNA‐independent heredity of TGS in *OsbZIP50* TGS lines. OsbZIP50 was detected by immunoblotting (IB) of total protein extracts obtained after root treatment with DTT (+) or without treatment (−). (a) T1 roots. Presence of T‐DNA was examined by PCR amplification of the *HPT* coding region of T‐DNA. Open squares indicate *OsbZIP50* TGS lines. (b) T2 roots of lines without T‐DNA.

To find lines without trigger genes (T‐DNA) in T1 populations of *CNX*,* OsbZIP50* and *Glb‐1* TGS lines, we screened them for the absence of T‐DNA by PCR and then for TGS by immunoblotting. Numerous TGS lines without trigger genes were found in the T1 population of *CNX* TGS lines (Figure 3) and *OsbZIP50* TGS lines (Figure 4), but none were found in a large T1 population of *Glb‐1* TGS lines (Figure S3).

In some *CNX* TGS lines that carried no trigger gene, TGS in leaves was inherited at least until the third generation (T3) (Figure 3b,c), but TGS was abolished in the endosperm of lines without the trigger gene (Figure 3d,e). TGS was inherited in the second generation (T2) of some *OsbZIP50* TGS lines without the trigger gene (Figure 4b). On the other hand, almost all progenies of TGS lines harbouring the trigger gene retained TGS.

In post‐transcriptional gene silenced (PTGS) lines generated by the expression of double‐strand RNA corresponding to a 1‐kb region of *CNX* mRNA (Figure S4a), PTGS was associated with the presence of the trigger gene and was inevitably abolished by the removal of this gene by segregation (Figure S4b). Thus, only RdDM‐mediated TGS was inherited even in the absence of the trigger gene. The results of these experiments are summarized in Table S1.

**Figure 5:**
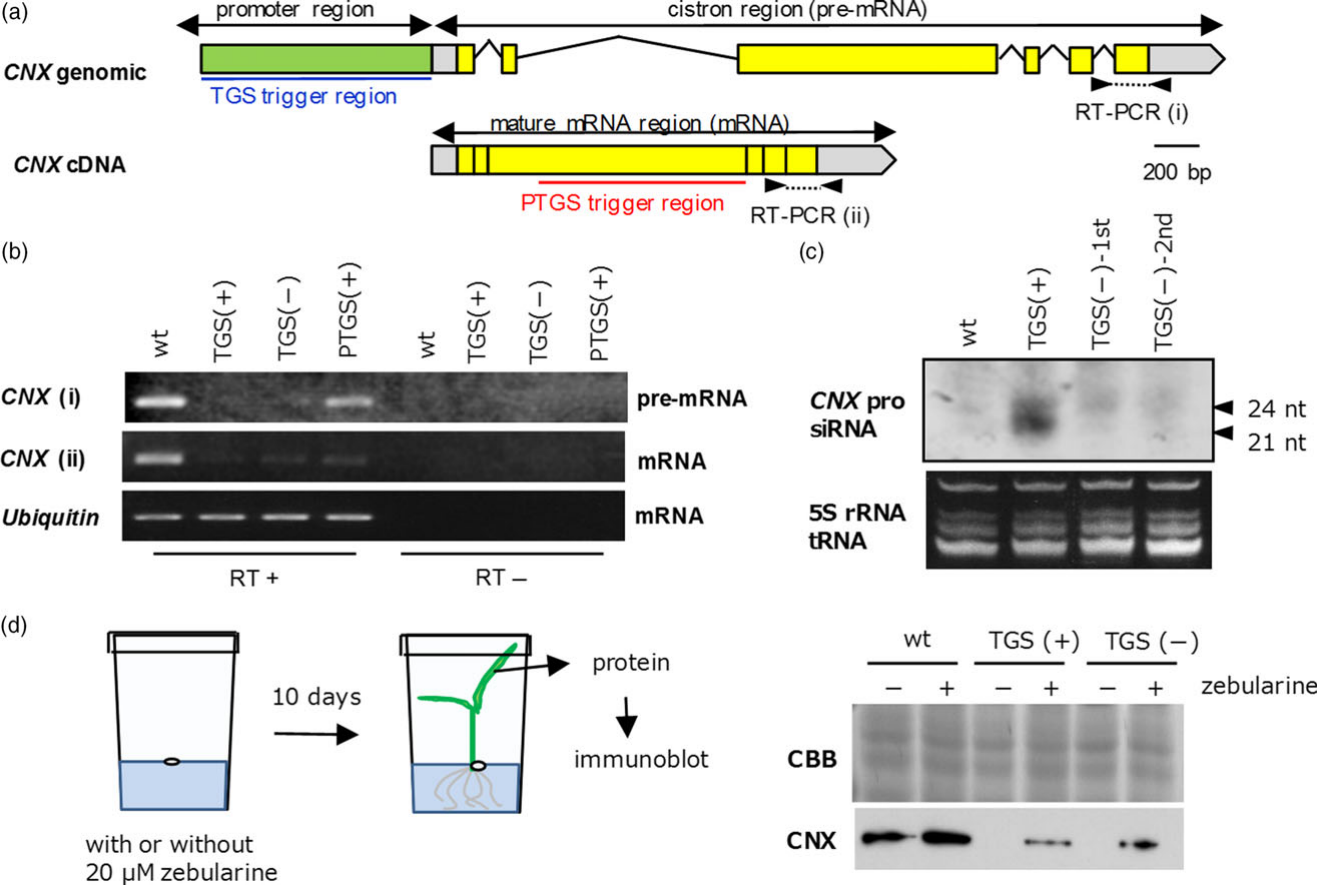
Molecular characteristics of *CNX* TGS lines with or without the trigger gene. (a) Genomic and cDNA structure of *CNX*. Trigger regions (1 kb each) for TGS (blue underline) and PTGS (red underline) and the regions for amplification of (i) premature mRNA and (ii) mature mRNA are shown. (b) Detection of premature and mature mRNA by RT‐PCR. RT+ and RT‐, with or without reverse transcription before PCR, respectively. Ubiquitin mRNA is shown as a loading control. (c) Northern blot analysis to detect siRNA. TGS trigger region of *CNX* was used as a probe. 1st and 2nd are number of generations after the removal of the trigger gene. (d) Scheme of rice seedling treatment with zebularine (a demethylation reagent) and immunoblot analysis of total protein extracts from leaves. In (b–d), (+) indicates the presence and (‐) the absence of the trigger gene (T‐DNA); wt, wild type. CBB, Coomassie Brilliant Blue (loading control).

To investigate TGS of the *CNX* gene, we examined the *CNX* transcripts in a *CNX* TGS line harbouring the trigger gene, a *CNX* TGS line lacking the trigger gene (2nd generation from the removal of the trigger gene) and a *CNX* PTGS line. We expected that transcription would be suppressed in TGS, whereas synthesized transcripts would be degraded in PTGS. Using RT‐PCR, we amplified premature or mature *CNX* mRNA from leaves (Figure 5a,b). Because premature mRNA before RNA splicing still contains intron, RT‐PCR using primer set consist of intron and coding regions (Figure 5a) can be detected the PCR products if transcription is occurred. As shown in Figure 5b, premature mRNA containing an intron was clearly detected in nontransgenic plants and *CNX* PTGS lines but not in *CNX* TGS lines. On the other hand, mature mRNA after splicing was clearly detected in nontransgenic plants only. These results confirmed our expectation that the transcription of *CNX* in TGS lines would be repressed, whereas its transcription in *CNX* PTGS lines would be maintained but *CNX* transcripts would be post‐transcriptionally degraded.

Because RdDM‐mediated TGS has a close correlation with siRNA derived from dsRNAs, we attempted to detect such siRNA in *CNX* TGS lines by Northern hybridization using the 1‐kb *CNX* promoter region to induce the TGS as a probe. As shown in Figure 5c, a strong signal corresponding to 21‐ to 24‐nt siRNAs was observed in a TGS line harbouring the trigger gene, whereas only a faint signal was observed in a TGS line lacking the trigger gene.

### The relationship between TGS and methylation level of promoter region

We abolished TGS by treatment with the demethylating reagent zebularine (Figure 5d) (Baubec *et al*., 2009). Seeds of *CNX* TGS lines were germinated on MS solid medium containing 20 μm zebularine for 10 days at 28 °C (16 h light/8 h dark). The CNX protein was detected in the leaves of TGS lines after treatment with zebularine but not in untreated TGS lines.

**Figure 6:**
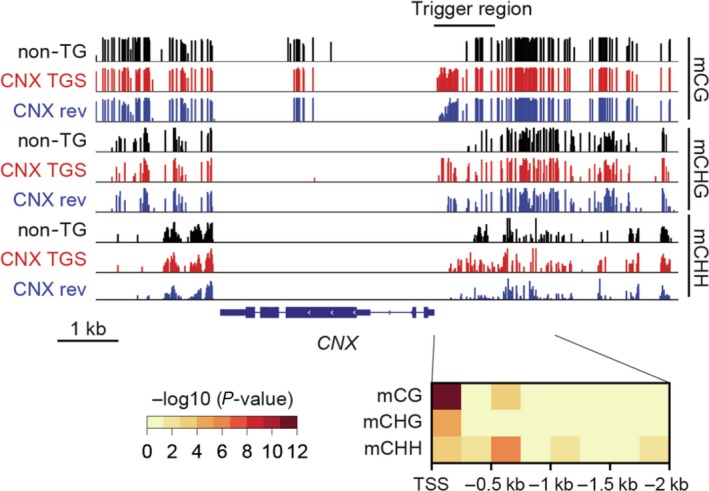
Methylation levels in the *CNX* promoter region in a line with preserved TGS and a line with TGS lost after the removal of the trigger gene. Top panel shows a snapshot of the methylation levels in each context around the *CNX* genes of control nontransgenic (non‐TG), *CNX* TGS and *CNX* revertant (CNX rev) lines. Methylation level at each site is indicated by tick height. The bottom panel shows *P*‐values (Fisher's exact test) for differential methylation in each context between *CNX* TGS and *CNX* rev.

The degree of cytosine methylation at CG, CHG and CHH (where H indicates A, T or C) in rice plants regenerated from callus without *Agrobacterium* infection (control), a *CNX* TGS line (T2) without the trigger gene, and a *CNX* TGS revertant line (T2) was investigated by bisulphite sequencing of the *CNX* promoter region (Figure 6). A statistically significant difference between the TGS and revertant lines was detected in the methylation levels of CG, CHG and CHH between −250 and −1 bp of the *CNX* promoter, especially, *P*‐value in CG and CHG showed less than 10^−4^ and 10^−10^. As expected, demethylation occurred more frequently in the revertant line. These results suggest that CG and CHG methylation may be sufficient for TGS of *CNX*, similar to VIGS‐induced TGS in *Arabidopsis* (Bond and Baulcombe, 2015).

Based on the result of bisulphite sequencing, we newly produced transgenic rice plants expressing dsRNA between the −250 and 3 bp region of *CNX* promoter (*CNX* TGS [−250 to 3]). As a result, TGS could be induced with similar rate to *CNX* TGS lines expressing the −1003 to 3 bp region of the promoter (*CNX* TGX [−1003 to 3]; Table 2). However, TGS levels in *CNX* TGS lines [−250 to 3] tended to be weaker than those in *CNX* TGS lines [−1003 to 3] (Table 2), suggesting that inducing DNA methylation within longer region of the promoter can induce TGS more effectively than that within shorter region. These results suggest that degree of participation of cytosine methylation may be different between the induction and abolishment of TGS.

**Table 2:** Size effect of *CNX* promoter region on transcriptional gene silencing induction rate and strength

Promoter region to induce TGS	No or faint signal in immunoblot	Weak signal in immunoblot	TGS plants/regenerated plants (%)
3 ˜ −1003 bp	38	6	44/66 (66.7%)
3 ˜ −250 bp	11	25	36/50 (72.0%)

### Induction of TGS of a gene family member

**Figure 7:**
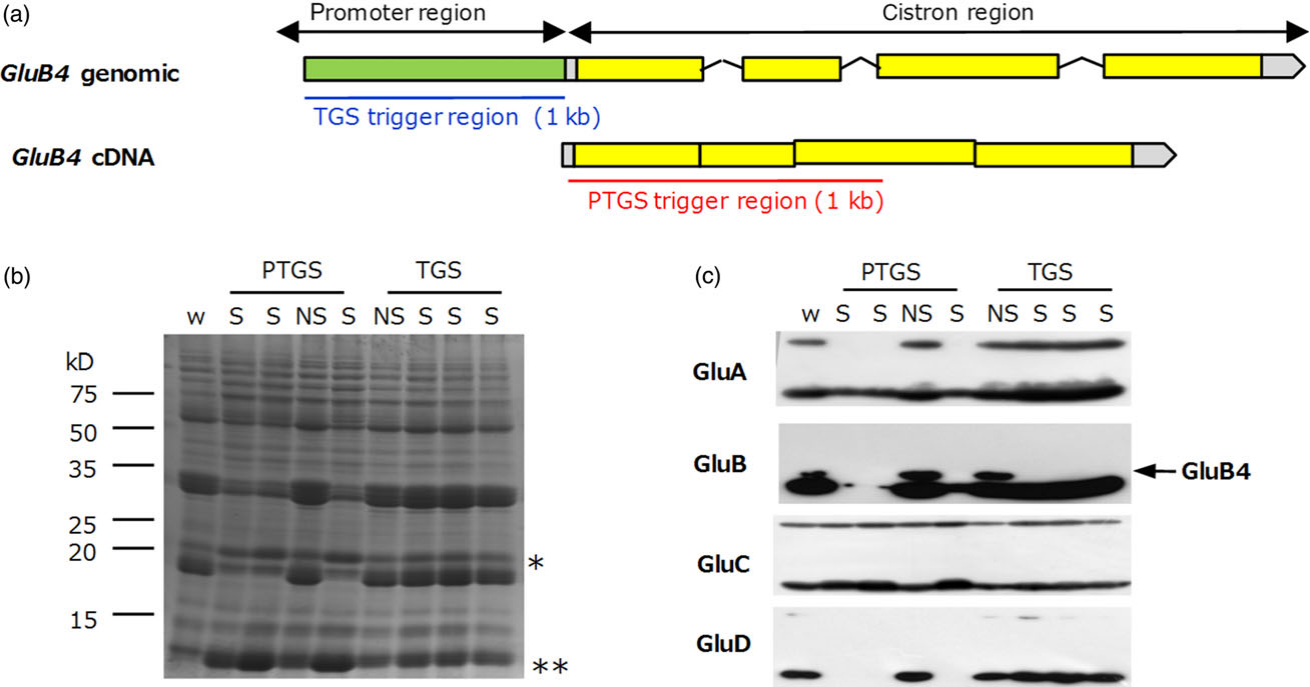
Comparison of PTGS and TGS specificity towards a target gene. (a) Structure of the *GluB4* gene and its transcript (cDNA). Target regions used to induce *GluB4* silencing are underlined (blue, TGS; red, PTGS). (b) Coomassie Brilliant Blue‐stained SDS‐PAGE gel showing seed proteins derived from PTGS and TGS lines (four seeds per line were analysed). w, wild type; S, silenced; NS, not silenced. * globulin; ** prolamins. (c) Immunoblot analysis of the same samples as in panel (b). Anti‐GluA antibody reacts with GluA1 and GluA2; anti‐GluB antibody reacts with GluB1, GluB2 and GluB4; anti‐GluC and GluD antibodies are specific. Upper bands, glutelin precursors; lower bands, glutelin acidic subunit.

Finally, we compared the specificity of the *GluB4* gene silencing between TGS and PTGS. The *GluB4* TGS line was produced by expression of dsRNA derived from a 1‐kb region of its promoter, whereas the *GluB4* PTGS line was produced by expression of dsRNA derived from its coding region (Figure 7a). Glutelins constitute a multigene family consisting of *GluA* (*GluA1*–*GluA3*), *GluB* (*GluB1*–*GluB4*), *GluC* and *GluD* (Kawakatsu *et al*., 2009). Sequence similarity among the coding regions of these genes is higher than that among their promoter regions (Table S2). Then, we considered that TGS can suppress only *GluB4* gene expression without co‐suppression of the other glutelin genes. The levels of these glutelins were compared in mature seed of the two lines. Only the level of the GluB4 protein was clearly decreased in the *GluB4* TGS line but the levels of some other glutelins (GluA1, GluA2, GluB1, GluB2 and GluD) were also decreased in the *GluB4* PTGS line (Figure 7). Only little changes were observed in other seed storage proteins such as prolamins, globulin and GluC in the TGS lines, whereas their levels were increased in the PTGS line by proteome rebalancing mechanisms as a result of a drastic decrease in the glutelin levels (Figure 7). These results suggest that TGS can be avoided co‐suppression among the gene family due to lower similarity of promoter regions compared with coding regions.

## Discussion

RNA‐directed DNA methylation‐mediated TGS is a promising NPBT approach, and there have been no reports of efficient RdDM‐induced TGS towards an endogenous gene in rice. TGS induction towards endogenous genes has been reported in *Arabidopsis*, potato, petunia and tobacco (Bond and Baulcombe, 2015; Deng *et al*., 2014; Heilersig *et al*., 2006; Ju *et al*., 2016; Kanazawa *et al*., 2011; Kasai *et al*., 2016; Kon and Yoshikawa, 2014). In this study, we generated rice plants with RdDM‐mediated TGS of six endogenous genes. Numerous TGS rice lines without the trigger gene were obtained among progeny of the *CNX* and *OsbZIP50* TGS lines, although the heredity of TGS was unstable. As expected, specificity of TGS towards the target gene was much higher than that of PTGS due to different similarity of sequences between the promoter and coding regions. In general, similarity of promoter region is lower than that of coding region among identical family genes such as rice glutelin genes. Although 5’ and 3’ UTRs may be also lower similarity than coding region, too short UTR would be difficult to apply to induce specific PTGS. Therefore, TGS would be useful for specific suppression of target genes that belong to large gene families.

In a previous study (Okano *et al*., 2008), RdDM of target promoter sequences was easily induced in seven endogenous rice genes, but TGS was not observed except for one gene, where TGS was incomplete. On the other hand, highly efficient induction of RdDM‐mediated TGS has been reported in stable rice transformants expressing GFP under the control of the CaMV 35S promoter (Okano *et al*., 2008). Which factors may have contributed to successful TGS induction towards endogenous genes in our study? First, the target promoter sequences were much longer in our study (800–1000 bp) than in the previous one (270–310 bp). Length of the RdDM region is reported to be one of the most important factors for efficient TGS induction (Otakagi *et al*., 2011). In the case of the CaMV 35S promoter, a shorter region (271 bp) may be sufficient, and TGS following virus‐induced RdDM towards an approximately 100‐bp region of the CaMV 35S promoter has been reported in transgenic tobacco (Otakagi *et al*., 2011). In this study, we could induce TGS of *CNX* gene by expression of 250 bp of its promoter region but the number of strong TGS line (means no signal was detected in immunoblot) was lower than *CNX* TGS lines expressing −1003 to 3 bp region of promoter (Table 2). This suggests that qualitative and quantitative *cis*‐elements locate between −250 and −1 bp and between −1000 and −250 bp upstream of the transcription start site of *CNX*, respectively, but crucial *cis*‐elements of genes analysed in Okano *et al*. (2008) did not locate within the target regions. It is also possible that inducing DNA methylation within longer region can affect chromatin states or distribution of repressive histone marks within longer region, resulting in effective TGS. Therefore, the use of long region of target promoter would be preferable for stable and strong TGS induction. Alternatively, the binding of transcription factors that positively regulate *CNX* is highly sensitive to DNA methylation, but those that regulate genes analysed in Okano *et al*. (2008) are not. It has been demonstrated that each transcription factor has distinct DNA methylation sensitivity (O'Malley *et al*., 2016). Additionally, the vector, in particular the spacer between the sense and antisense directions of the target sequences, may be another contributing factor. The spacer was derived from the GUS sequence in Okano *et al*., 2008; whereas the vector pZH2Bik (Kuroda *et al*., 2010) we used, contained the 2nd intron of the rice aspartic protease gene as a spacer. The use of an intron derived from a rice gene may enhance the generation of dsRNA and/or siRNA.

In our study, the efficiency of TGS induction varied (from 10% to 67%) among target genes. Not only strong TGS lines but also moderate and weak TGS lines were found. A major reason for this variability is the difference in the expression levels of the TGS trigger gene cassette caused by the difference in T‐DNA copy number or by positional effects. Higher expression of the trigger gene would yield more siRNA and induce RdDM more efficiently. Some features of the promoter region such as the number and distribution of cytosine residues may also be responsible for the difference in TGS efficiency. DNA methylation within the promoter region is often linked to gene repression because it inhibits binding of transcription factors (Medvedeva *et al*., 2013). DNA methylation may lead to chromatin modification, and *cis*‐elements in heterochromatic promoter regions become inaccessible to their cognate transcription factors.

DNA methylation within the promoter regions was well associated with TGS in *CNX*,* OsbZIP50* and *OsBiP1* lines (high TGS induction efficiency), but not in the *Glb‐1* line (low TGS induction efficiency; Figure 1 and Figure S1). These results suggest that differences in TGS induction efficiency depend on combinatorial effects of siRNA abundance, DNA methylation and chromatin modification. In this study, the level of cytosine methylation correlated well with TGS strength (Figures 2 and 6). In the future, we intend to investigate the relationships among the TGS induction rate, siRNA amounts and changes in chromatin modification.

Our study showed that 21‐ to 24‐nt siRNAs were generated from transcripts derived from trigger gene cassettes, resulting in RdDM‐mediated TGS. These siRNAs were likely generated from dsRNA by DCL proteins (Matzke *et al*., 2015). After the removal of the trigger gene, the siRNA levels were drastically decreased even in lines with preserved TGS. Because the expression of target genes in TGS lines harbouring T‐DNA was stably suppressed, the siRNA level may be a key factor for TGS preservation. In addition, the loss of DNA methylation within the promoter of *CNX* in the *CNX* TGS revertant line suggests active demethylation occurred within this region, because CG and CHG methylation can be maintained by distinct mechanisms other than RdDM. Thus, demethylation must be avoided to preserve TGS after trigger gene removal. Although further investigation is necessary in this regard, our TGS rice lines may be useful in solving the problems about stable preservation of TGS after trigger gene removal.

Transcriptional gene silencing of *Glb‐1* was associated with the presence of the trigger gene in endosperm tissue (Figures S3). The *CNX* TGS lines lacking the trigger gene showed TGS in leaves but not in the endosperm even though they were derived from the same seed (Figure 3a,d,e). One explanation is that endosperm cells are derived from a central cell that is generated through meiosis. Higher demethylation rate of cytosine residues has been observed in the central cell of the female gamete after meiosis than in other tissues, which is involved in genome imprinting (Gehring *et al*., 2006; Hsieh *et al*., 2009; Ibarra *et al*., 2012; Kawashima and Berger, 2014; Kinoshita *et al*., 2004). Bisulphite‐genome sequencing showed that levels of DNA methylation (CG, CHG and CHH) in central cells are lower than that in embryo cells (Hsieh *et al*., 2009), suggesting that demethylation in central cells does not have strict specificity towards genomic sequences. Therefore, DNA demethylation in the target promoter region may abolish TGS in endosperm cells, as TGS was abolished by zebularine treatment even in lines harbouring the trigger gene. Bisulphite sequencing showed a significant difference in the methylation levels of CG and CHG in the *CNX* gene promoter between lines with preserved TGS and revertant lines after the removal of the trigger gene. Thus, preservation of methylation of cytosine residues in the target promoter would be the most important factor for stable heredity of TGS after the removal of the trigger gene.

In this study, TGS rice lines with suppressed expression of six endogenous genes were generated by expressing dsRNA corresponding to their promoter regions. We demonstrated the trigger gene‐independent heredity of TGS. The development of a technique for stable preservation of TGS is a future challenge and would be necessary for applying RdDM‐mediated TGS in rice breeding. We believe that this will be possible because recent high‐precision genomewide omics studies have led to a rapid accumulation of information on genomewide landscapes of epigenetic modifications and the relationship between epigenetic modifications and plant growth regulation (Akman *et al*., 2014; Kawakatsu *et al*., 2016). The use of RdDM‐mediated TGS may be more advantageous in vegetatively propagated crops such as potato and apple than in seed‐propagated crops such as rice because the former do not need meiosis for self‐reproduction.

## Experimental procedures

### Plant materials

Rice (*Oryza sativa* L. cv. Kitaake) was used for transformation.

#### Vector construction and generation of transgenic rice

Approximately 1‐kb promoter regions of genes encoding calnexin (*CNX*, −1003 to 3 bp), OsbZIP50 (−965 to −1 bp), globulin (*Glb‐1*, −858 to −3 bp), glutelin B4 (*GluB4*, −997 to −1 bp), protein disulphide isomerase like 1‐1 (*PDIL1‐1*) and luminal binding protein 1 (*OsBiP1*, −1017 to −5 bp) were cloned into pCR‐Blunt II‐TOPO vector using a Zero Blunt TOPO cloning kit (Invitrogen). To generate the dsRNA targeted to each promoter region, these fragments were subcloned into the pZH2Bik binary vector (Kuroda *et al*., 2010), which includes the rice ubiquitin promoter, the 2nd intron of the rice aspartic protease (*RAP*) gene interposed between the target sequence in the sense and antisense direction (inverted target sequences), and the Nos terminator. Constructs are shown in Figure. 1a. Rice transformation was performed as described previously (Goto *et al*., 1999).

#### Total protein extraction and immunoblot analysis

Mature seed, leaves and roots were ground into a fine powder with a Multi‐Beads Shocker (Yasui Kikai). For protein extraction, 300 μL (10 mg of leaves and roots) or 500 μL (one seed) of extraction buffer [50 mm Tris‐HCl, 8 m urea, 4% SDS, 20% glycerol, 5% 2‐mercaptoethanol, 0.01% Bromophenol Blue] was added, and the samples were vortexed for 1 h at room temperature. The mixture was centrifuged at 12,000 × ***g*** for 10 min at room temperature, and the supernatant (protein extract) was decanted into a new tube. Total protein (2 μL of seed extract, 5 μL of leaf and root extracts) was electrophoresed in 12% SDS‐PAGE gels and subjected to immunoblot analysis. Antibodies against calnexin, globulin, OsbZIP50, PDIL1‐1, GluA, GluB, GluC and GluD were used as described previously (Takagi *et al*., 2006; Wakasa *et al*., 2011; Yasuda *et al*., 2009).

#### Reverse transcription (RT)‐PCR

Total RNA was extracted using an RNeasy Plant Mini kit (Qiagen). An RT reaction was performed with ReverTra Ace qPCR RT Master Mix with gDNA Remover (Toyobo). Briefly, genomic DNA was removed, and total RNA (0.5 μg) was used in an RT reaction as described in the manufacturer's protocol. RT products (1 μL) were subjected to PCR using KOD FX Neo (Toyobo). Primer sets used in this study are listed in Table S3.

#### Quantitative PCR (qPCR)

Genomic DNA (200 ng) was digested overnight with the methylation‐sensitive restriction enzyme *Hap*II in a 20‐μL reaction mixture. A 1 μL aliquot of this mixture was subjected to qPCR amplification (98 °C for 2 min, followed by 40 cycles of 98 °C for 10 s, 60 °C for 10 s and 68 °C for 30 s) with KOD SYBR qPCR Mix (Toyobo) and a ViiA7 real‐time PCR system (Applied Biosystems) according to the manufacturers’ protocols. The primer set (*CNX*) is shown in Table S3. The methylation level was determined from the relative ΔCt differences from wild‐type genomic DNA without *Hap*II digestion. Means and SD were calculated from three replicates.

#### MethylC‐seq

DNA was isolated from rice leaves using the CTAB method (Murray and Thompson, 1980). Genomic DNA (1 μg) was used for MethylC‐seq library preparation as described previously (Urich *et al*., 2015). Samples were sequenced on an Illumina HiSeq 4000 instrument at the Vincent J. Coates Genomics Sequencing Laboratory at UC Berkeley. Read mapping and base calling were performed with the methylpy pipeline (Schultz *et al*., 2015; https://bitbucket.org/schultzmattd/methylpy), except that the reads were mapped against the C‐to‐T converted IRGSP‐1.0 reference genome. The bisulphite nonconversion rate was calculated as the total number of cytosine base calls divided by the total coverage at cytosine positions in the unmethylated chloroplast genome. For differential methylation analysis, a 2‐kb promoter region of the *CNX* gene was split into 8 bins, and the methylation levels were compared by Fisher's exact test.

### Accession numbers

Gene locus IDs in RAP‐DB (http://rapdb.dna.affrc.go.jp/) are listed in Table S4.

## Conflict of interest

The authors declare no conflict of interest.

## Supporting information


**Figure S1** Methylation analysis using restriction enzyme digestion in transgenic rice lines harbouring trigger genes to induce TGS of *CNX*,* OsbZIP50*, or *Glb‐1*.

**Figure S2** TGS of the *OsBiP1* gene is developmentally lethal.

**Figure S3** TGS of the *Glb‐1* gene in T1 and T2 generations with or without trigger gene.

**Figure S4** Analysis of the relationship between the presence of the trigger gene and PTGS.

**Table S1** Relationship between the presence of T‐DNA (trigger gene) and gene silencing in progeny of *CNX* TGS, *CNX* PTGS, and *OsbZIP50* TGS lines
**Table S2** Nucleotide sequence similarity among the coding regions (PTGS target sequences) and promoter regions (TGS target sequences) of rice glutelin genes
**Table S3** Gene‐specific primers used in this study
**Table S4** Gene names and corresponding locus IDs
