# Supplementary figures and images for: Transgene‐independent heredity of RdDM‐mediated transcriptional gene silencing of endogenous genes in rice
Source: Plant Biotechnol J. 2018 May 30;16(12):2007–15. doi: 10.1111/pbi.12934 (PMC6230945; doi:10.1111/pbi.12934)

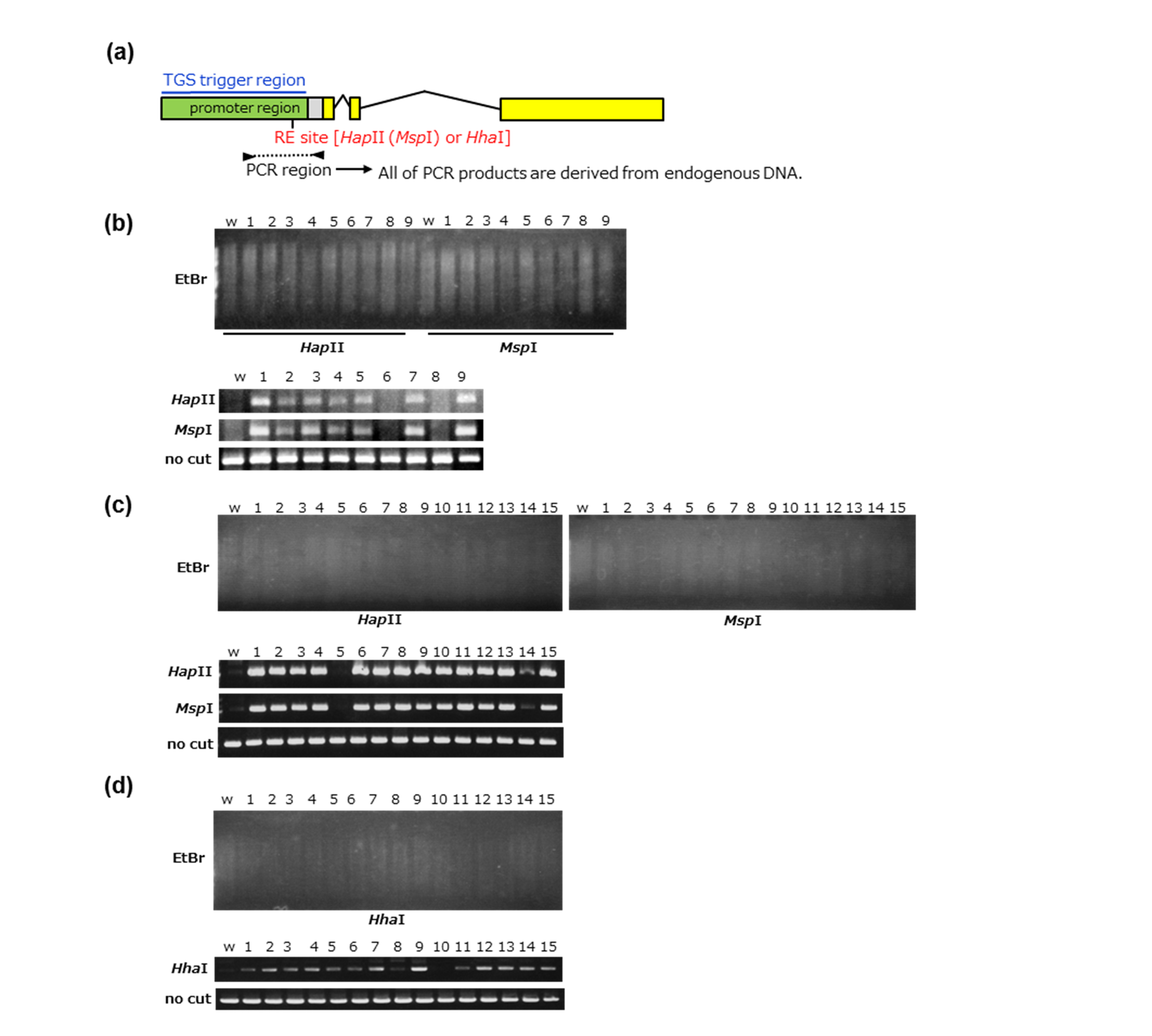

Supplement: Supplementary file 1 — Figure S1 Methylation analysis using restriction enzyme digestion in transgenic rice lines harbouring trigger genes to induce TGS of CNX, OsbZIP50, or Glb‐1. [file PBI-16-2007-s004.tif]

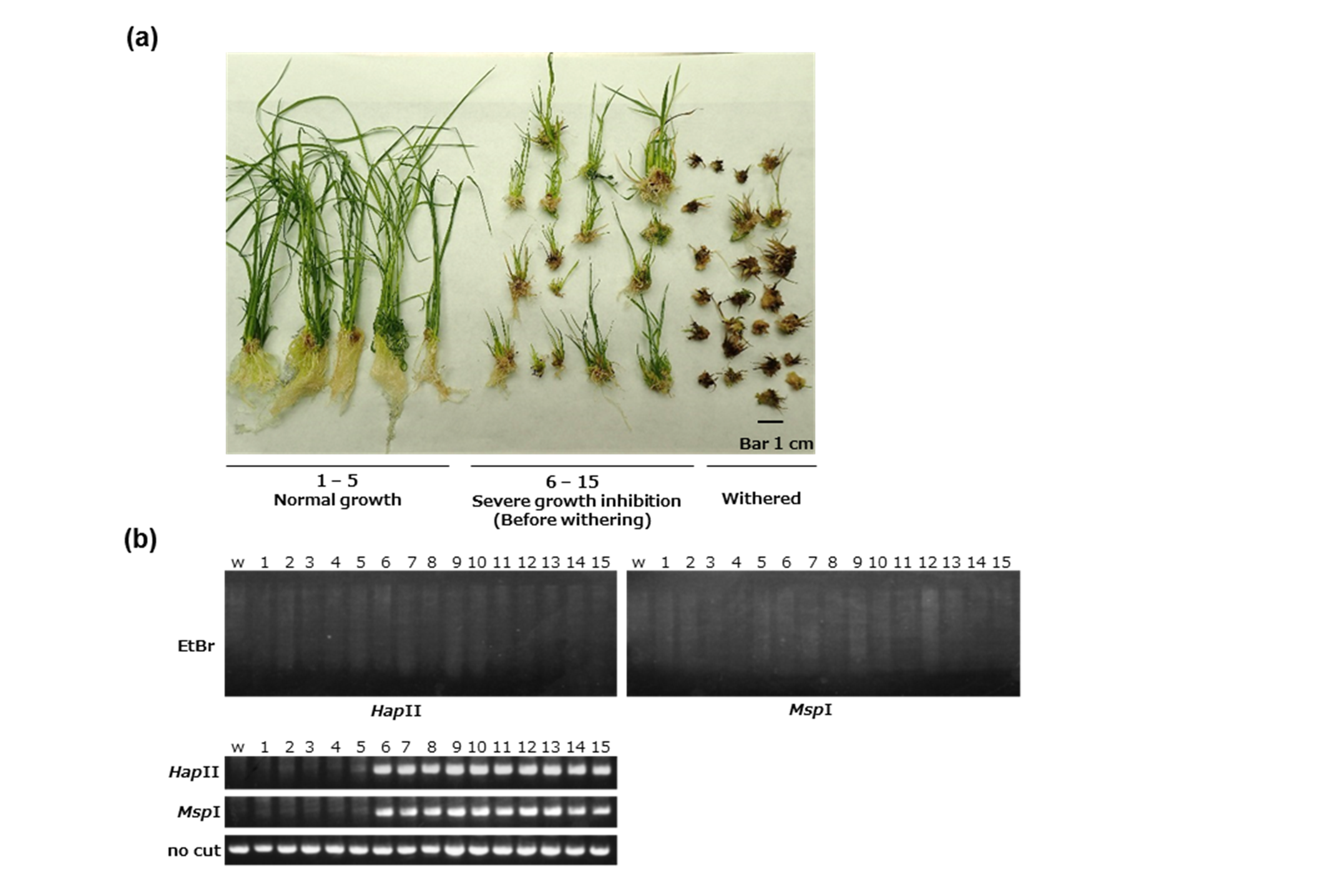

Supplement: Supplementary file 2 — Figure S2 TGS of the OsBiP1 gene is developmentally lethal. [file PBI-16-2007-s003.tif]

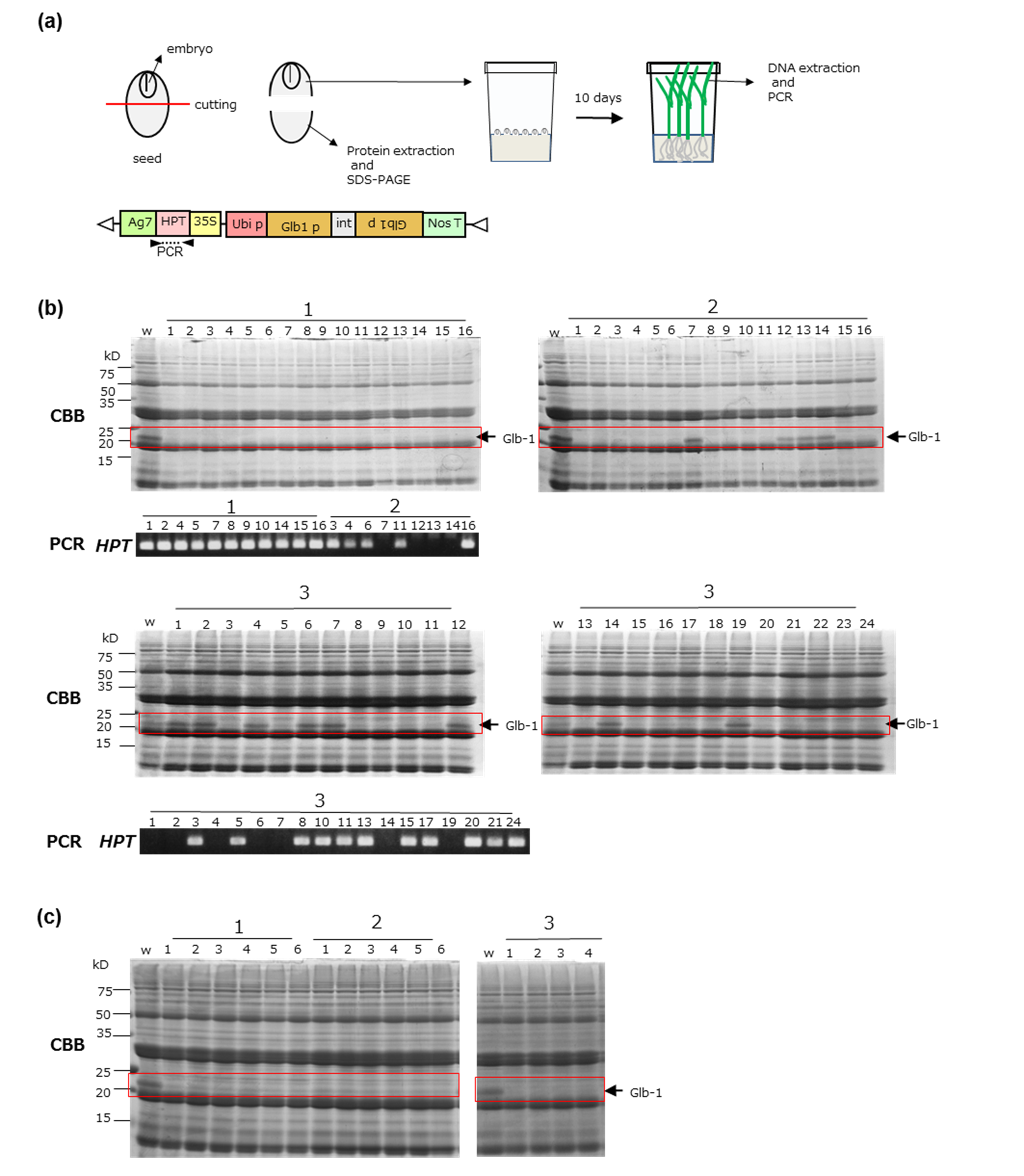

Supplement: Supplementary file 3 — Figure S3 TGS of the Glb‐1 gene in T1 and T2 generations with or without trigger gene. [file PBI-16-2007-s005.tif]

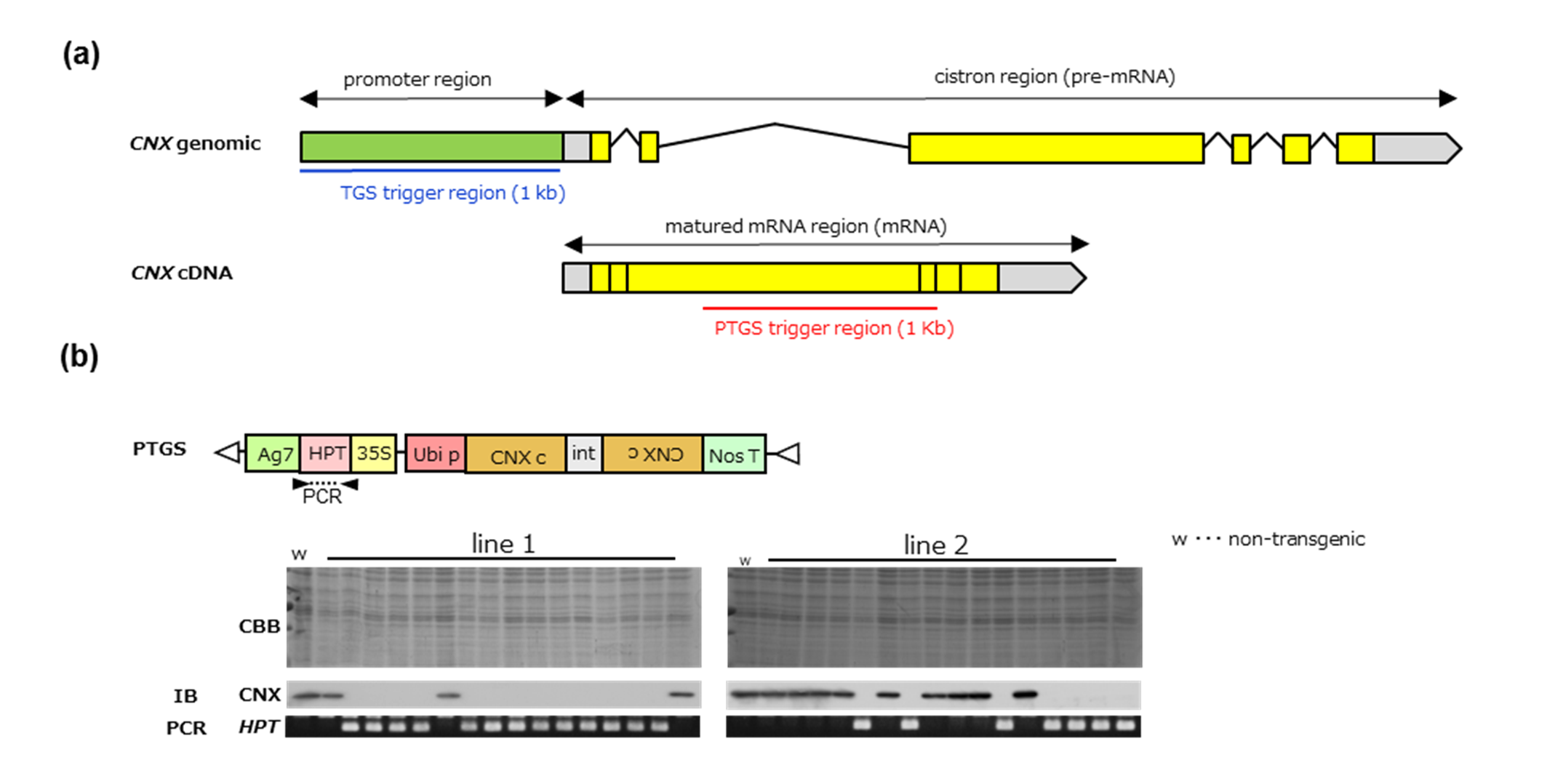

Supplement: Supplementary file 4 — Figure S4 Analysis of the relationship between the presence of the trigger gene and PTGS. [file PBI-16-2007-s002.tif]
