# Supplementary material for: Transgene‐independent heredity of RdDM‐mediated transcriptional gene silencing of endogenous genes in rice
Source: Plant Biotechnol J. 2018 May 30;16(12):2007–15. doi: 10.1111/pbi.12934 (PMC6230945; doi:10.1111/pbi.12934)
Supplement: Supplementary file 5 — Table S1 Relationship between the presence of T‐DNA (trigger gene) and gene silencing in progeny of CNX TGS, CNX PTGS, and OsbZIP50 TGS lines Table S2 Nucleotide sequence similarity among the coding regions (PTGS target sequences) and promoter regions (TGS target sequences) of rice glutelin genes Table S3 Gene‐specific primers used in this study Table S4 Gene names and corresponding locus IDs [file PBI-16-2007-s001.docx]

**Supplementary Tables**

Table S1 Relationship between the presence of T-DNA (trigger gene) and gene silencing in progeny of *CNX* TGS, *CNX* PTGS, and *OsbZIP50* TGS lines.

| **TGS calnexin (*CNX*) T0 → T1** | **T-DNA (+)**  **TGS / number of individuals** | **T-DNA (-)**  **TGS / number of individuals** |
| --- | --- | --- |
| line 1 | 0/0 | 2/2 |
| line 9 | 2/2 | 2/2 |
| line 12 | 0/0 | 1/1 |
| line 25 | 2/2 | 1/3 |
| line 44 | 1/1 | 3/6 |
| line 50 | 2/2 | 1/1 |
| line 57 | 3/3 | 1/1 |
| ***CNX* T1 → T2** |  |  |
| 12-1 | 8/8 | 0/0 |
| 12-2 | 8/8 | 0/0 |
| 12-3 | 0/0 | 8/8 |
| 12-4 | 0/0 | 8/8 |
| 25-1 | 8/8 | 0/0 |
| 25-2 | 8/8 | 0/0 |
| 25-3 | 0/0 | 7/8 |
| 50-1 | 8/8 | 0/0 |
| 50-2 | 8/8 | 0/0 |
| 50-3 | 0/0 | 8/8 |
| 57-1 | 0/0 | 7/8 |
| ***CNX* T2 → T3** |  |  |
| 12-3-1 | 0/0 | 0/4 |
| 12-3-2 | 0/0 | 0/4 |
| 12-3-3 | 0/0 | 0/4 |
| 12-4-1 | 0/0 | 0/4 |
| 25-3-1 | 0/0 | 3/3 |
| 25-3-2 | 0/0 | 3/3 |
| 25-3-3 | 0/0 | 3/3 |
| 50-3-1 | 0/0 | 3/3 |
| 50-3-2 | 0/0 | 3/3 |
| 50-3-3 | 0/0 | 3/3 |
| 57-4-1 | 0/0 | 1/2 |
| 57-4-2 | 0/0 | 0/2 |
| 57-4-3 | 0/0 | 4/4 |
| 57-4-4 | 0/0 | 0/2 |
| **PTGS *CNX* T0 → T1** | **T-DNA (+)**  **PTGS / number of individuals** | **T-DNA (-)**  **PTGS / number of individuals** |
| line 1 | 13/13 | 0/4 |
| line 2 | 7/7 | 0/10 |
| line 3 | 13/13 | 0/3 |
| **TGS**  ***OsbZIP50* T0 → T1** | **T-DNA (+)**  **TGS / number of individuals** | **T-DNA (-)**  **TGS / number of individuals** |
| line 1 | 1/1 | 0/2 |
| line 2 | 2/2 | 0/1 |
| line 3 | 2/2 | 1/1 |
| line 4 | 3/3 | 0/0 |
| line 6 | 2/3 | 0/0 |
| line 9 | 3/3 | 0/0 |
| line 11 | 3/3 | 0/0 |
| line 13 | 1/3 | 0/0 |
| line 16 | 3/3 | 0/0 |
| line 18 | 3/3 | 0/0 |
| line 19 | 2/2 | 0/1 |
| line 21 | 3/3 | 0/0 |
| line 23 | 2/2 | 0/0 |
| line 24 | 2/2 | 1/1 |
| line 25 | 1/1 | 1/2 |
| line 26 | 2/2 | 1/1 |
| line 27 | 2/2 | 0/1 |
| line 31 | 3/3 | 0/0 |
| line 33 | 1/1 | 2/2 |
| line 37 | 2/2 | 1/3 |
| line 39 | 3/3 | 0/0 |
| line 40 | 2/2 | 0/1 |
| line 41 | 3/3 | 0/0 |
| line 42 | 2/2 | 0/0 |
| line 45 | 2/2 | 0/1 |
| line 46 | 3/3 | 0/0 |
| ***OsbZIP50* T1 → T2** |  |  |
| 33-1 | 0/0 | 3/3 |
| 33-2 | 0/0 | 3/3 |
| 33-3 | 3/3 | 0/0 |
| 33-4 | 0/0 | 3/3 |
| 33-5 | 0/0 | 0/3 |
| 33-6 | 0/0 | 0/3 |

Table S2. Nucleotide sequence similarity among the coding regions (PTGS target sequences) and promoter regions (TGS target sequences) of rice glutelin genes.

Coding regions

|  | GluA1 | GluA2 | GluB1 | GluB2 | GluB4 | GluC | GluD |
| --- | --- | --- | --- | --- | --- | --- | --- |
| GluA1 | 100 | 95 | 65 | 66 | 67 | 54 | 61 |
| GluA2 |  | 100 | 65 | 65 | 65 | 54 | 63 |
| GluB1 |  |  | 100 | 91 | 83 | 54 | 72 |
| GluB2 |  |  |  | 100 | 83 | 54 | 71 |
| GluB4 |  |  |  |  | 100 | 52 | 72 |
| GluC |  |  |  |  |  | 100 | 54 |
| GluD |  |  |  |  |  |  | 100 |

Promoter regions (1 kb)

|  | GluA1 | GluA2 | GluB1 | GluB2 | GluB4 | GluC | GluD |
| --- | --- | --- | --- | --- | --- | --- | --- |
| GluA1 | 100 | 72 | 49 | 47 | 48 | 44 | 47 |
| GluA2 |  | 100 | 48 | 54 | 47 | 44 | 48 |
| GluB1 |  |  | 100 | 70 | 56 | 45 | 51 |
| GluB2 |  |  |  | 100 | 54 | 46 | 49 |
| GluB4 |  |  |  |  | 100 | 46 | 47 |
| GluC |  |  |  |  |  | 100 | 44 |
| GluD |  |  |  |  |  |  | 100 |

Table S3. Gene-specific primers used in this study.

| Purpose | Forward primer (5′ – 3′) | Reverse primer (5′ – 3′) |
| --- | --- | --- |
| *CNX* methylation analysis and qPCR | TTTCGGGTCGTGAGCACAGCGTGG | AGGAGAAGAAGAACAGAGCTGC |
| *OsbZIP50* methylation analysis | TGTTATTAATATATATTTCGGTC | AGGTCGGCGAAGAACTCTACATCC |
| *Glb-1* methylation analysis | CGCCTGGAGGGAGGAGAGGGGAGAG | CTCTTTTGTTTGTTGGTGATGAACTG |
| *OsBiP1* methylation analysis | CAATTACACATTGGGCCTGTTGAG | CTTCAAGTGAGTTTGAGTTTTATGG |
| *CNX* premature RNA amplification | TGATCGTAAGATTTGCATTCCCTAG | ATCTGCAGGGAGCATAGGCGACATG |
| *CNX* mature RNA amplification | TCCTGTTCGGCGGCAAGAAGCCAGC | ATCTGCAGGGAGCATAGGCGACATG |
| *HPT* for detection of T-DNA | GCGACGTCTGTCGAGAAGTTTCTG | TTCGGTTTCAGGCAGGTCTTGC |

Table S4. Gene names and corresponding locus IDs.

| Gene name | *RAP-DB locus number |
| --- | --- |
| *CNX* | Os04g0402100 |
| *OsbZIP50* | Os06g0622700 |
| *Glb-1* | Os05g0499100 |
| *PDIL1-1* | Os11g0199200 |
| *OsBiP1* | Os02g0115900 |
| *GluB4* | Os02g0268300 |
| *GluA1* | Os01g0762500 |
| *GluA2* | Os10g0400200 |
| *GluB1* | Os02g0249800, Os02g0249900 |
| *GluB2* | Os02g0249600 |
| *GluC* | Os02g0453600 |
| *GluD* | Os02g0249000 |
| *RAP* | Os05g0567100 |

*http://rapdb.dna.affrc.go.jp/
